# Global Public Health Database Support to Population-Based Management of Pandemics and Global Public Health Crises, Part II: The Database

**DOI:** 10.1017/S1049023X20001363

**Published:** 2020-10-22

**Authors:** Frederick M. Burkle, David A. Bradt, Joseph Green, Benjamin J. Ryan

**Affiliations:** 1. Professor (Ret.) Senior Fellow and Scientist, Harvard Humanitarian Initiative, Harvard University, T.H. Chan School of Public Health, Cambridge, Massachusetts, USA; 2. Global Scholar, Woodrow Wilson International Center for Scholars, Washington, DC, USA; 3. Dept of International Health, Johns Hopkins School of Public Health, Baltimore, Maryland, USA; 4. Acting Director of Applied Science, Pacific Disaster Center, Hawaii, USA; 5. Clinical Associate Professor, Department of Environmental Science, Baylor University, Waco, Texas, USA

**Keywords:** global public health, pandemics, population-based management, public health, triage, AI, artificial intelligence, COVID-19, coronavirus disease 2019, GPH, global public health, PH, public health, PBM, population-based management, PBMT, population-based management team, WHO, World Health Organization

## Abstract

This two-part article examines the global public health (GPH) information system deficits emerging in the coronavirus disease 2019 (COVID-19) pandemic. It surveys past, missed opportunities for public health (PH) information system and operational improvements, examines current megatrend changes to information management, and describes a new multi-disciplinary model for population-based management (PBM) supported by a GPH Database applicable to pandemics and GPH crises.

## Introduction

The 2019 novel coronavirus (SARS-CoV-2, 2019-nCoV) disease (COVID-19) has become the first pandemic of the 21^st^ century. Viral contributory causes include high virus shedding from upper respiratory tract secretions, symptomatic and pre-symptomatic virus transmission, reproduction ratio estimates up to 3.58,^1^ little effective antiviral therapy, and no effective vaccine. Health system and community contributory causes include wide-spread community seeding through unrestricted air travel, inadequate implementation of non-pharmacologic control measures, limited contact tracing and viral diagnostic capabilities, overwhelmed clinical care facilities, and threat minimization by elected officials.

These contributory causes have drawn attention to potential remedies for pandemic management by public health (PH) authorities. Part I reviewed past missed opportunities for PH information systems and operational improvements, then examined important PH megatrends heralding future advances in pandemic management—evidence-based decision making, data literacy, ascent of PH information services, and ascent of remote management. Part I also reviewed the emergence of population-based management (PBM) of health crises. A multi-disciplinary model led by PH professionals is required to ensure data driven decisions can be made that reflect the PH needs and risks of local communities. This approach builds on the evolution of disaster management systems, which are used in most countries across the world, to regularly deal with local, state, national, and international crises. This article examines database requirements and modalities of functioning for PBM teams (PBMTs).

## Digital Technology

Digital technologies may help perform a wide range of functions in pandemic management, as summarized in Table 1.^2^ Health authorities in some countries have attributed their disease control successes to early adoption of such technology.^3-5^

**Table 1.** Applications of Digital Technology in Pandemics

Function	Technology	Early Adopters
Infection Screening	Digital thermometers, infrared thermal cameras, web-based tools	China, Iceland, Singapore, Taiwan
Contact Tracing	GPS, mobile phone apps, facial recognition technology	China, Germany, Singapore, South Korea
Quarantine and Isolation	GPS, mobile phone apps, surveillance of mobile devices for voluntary or involuntary tracking	China, Iceland, South Korea, Taiwan, Australia
Clinical Management	Lab test pooling, telemedicine, video-conferencing, AI algorithms for clinical outcome prediction	Australia, Canada, China, USA
Medical Logistics	Mobile apps for transactions, barcode scanners for commodities, robotics, and drones for delivery	US, China
Epidemic/Pandemic Tracking	Data dashboards, real-time data transmitted by smartphones and PDAs	China, Singapore, Sweden, USA

Source: Adapted from Whitelaw.^2^

Abbreviations: AI, artificial intelligence; PDA, personal digital assistant.

Nonetheless, major issues have arisen on data oversight, privacy, protection of personally identifiable health information, and redress for errors that implementing jurisdictions must confront. The scientific community has seen retractions of papers published in *The Lancet* and *The New England Journal of Medicine* due to post-publication concerns over provenance of proprietary data and inconsistencies from electronic health records.^6^ This experience is cautionary about the importance of data source/origin, data audit trails, and oversight in managing such datasets.

## Core Databases

Core Databases for PBM are characterized below in three overriding domains:

1. Background Information (Table 2) describes the affected population pre-pandemic—demography, access to essential services, health status, and socioeconomic status.
2. Clinical Case and Virological Data (Table 3) encompasses case definitions, case identification and outcomes, and clinical dynamics (case-fatality rates, death rates, and time course to death).
3. PH Control Measures and Consequences (Table 4) encompasses details of contact tracing, quarantine measures, travel restrictions, and all epidemic modelling.

**Table 2.** Datasets and Visualizations Pre-Pandemic

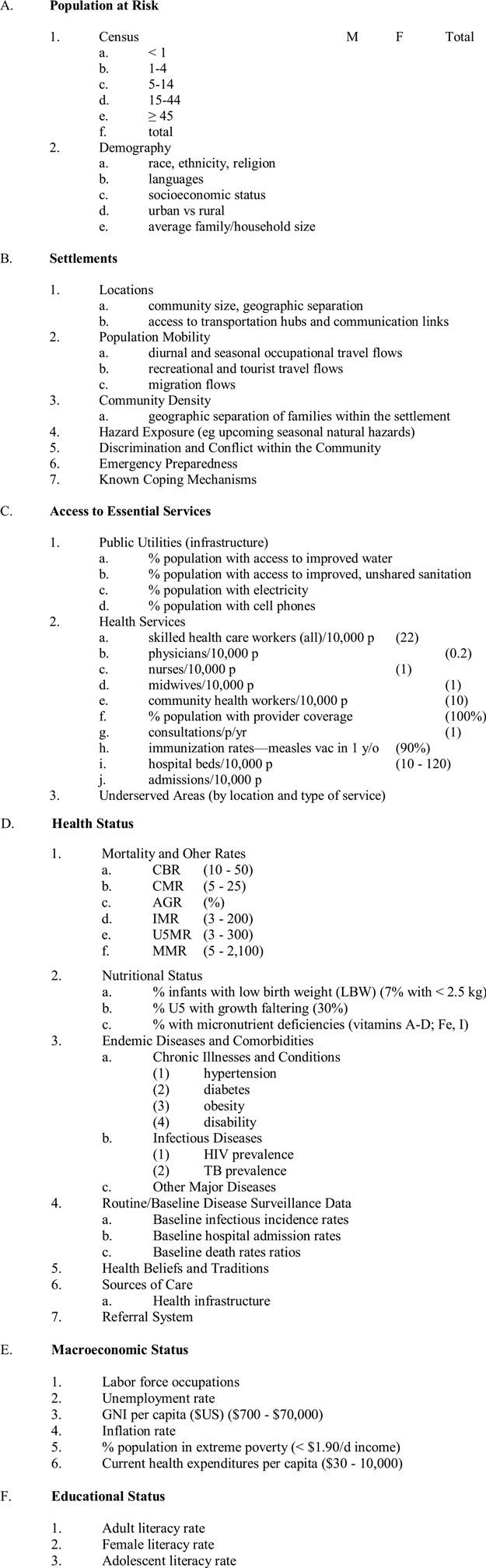

**Table 3.** Datasets and Visualizations Intra-Pandemic

Clinical and Virological Data: **Clinical Data** Case Definitions (suspected, probable, confirmed) Severe Cases (admissions, ICU cases, ventilated cases) Age/gender/race data Comorbidities Clinical course, complications, and outcomes Data on use of antivirals, antibodies, and vaccines Discharge diagnoses Causes of death Clinical Dynamics—population morbidity and mortality rates, age & gender-specific CFRs, time course to death Hotspot Definitions—cases linked temporally or geographically triggering further outbreak investigation and/or control measures **Virological Data** Sentinel Site Testing Confirmatory testing for new cases in the community meeting the surveillance definition (before sustained community transmission is identified) daily new positive cases 14 day running average Confirmatory testing for hospital admissions and unexplained deaths that are compatible with the disease Community seroprevalence testing once community transmission is established On-demand individual testing (depending on resource availability, may be targeted at vulnerable groups) Antiviral Sensitivity Testing

**Table 4.** Datasets and Visualizations Intra-Pandemic

Modelling, Control Measures, and Impact: **Epidemic Modelling Data** Population sizes and subgroups at risk Compartment models Spot maps (geolocated plots of disease outbreaks) Kinetics models of transmission dynamics—incubation period, reproduction ratio, contacts per case Epidemic curves Epidemic projections showing impact of non-pharmaceutical mitigation measures **Public Health Control Measures** Travel restrictions Arrival testing Contact tracing effectiveness Quarantine requirements (site specification, duration, monitoring), social acceptance of quarantine measures, overall adequacy of quarantine Social distancing recommendations Mask recommendations Restrictions on social gatherings Lockdown onset, duration Population sizes & community densities of populations affected **Socio-Economic Impact Data** Displacements Unemployment rates Evictions Business closures Direct costs in health care Indirect costs of business losses Government expenditures for unemployed persons and distressed businesses

## The Database Challenge

The technical standards for disease surveillance and surveillance systems comprise an extensive literature. The Centers for Disease Control and Prevention (CDC; Atlanta, Georgia USA) published its first guidelines in 1988, which were re-issued in 2001.^7,8^ The World Health Organization (WHO; Geneva, Switzerland) Program on Disease Control in Humanitarian Emergencies published numerous adaptations of communicable disease and surveillance guidelines for epidemic-prone diseases in natural disasters and complex emergencies through the 2000s. Recent emerging infectious diseases such as Ebola have prompted additional reviews of data needed for outbreak evaluation culminating in descriptions of an emerging science of outbreak analytics.^9,10^

Taking into account the rate of global population growth, it was predicted in 2019 there would be 1.7MB data created every second for every person on Earth, bringing forth new data problems, “most notably where and how to store it.”^9^ With unprecedented movement of migrants and refugees from the Middle East, compounded by massive relocations of urban populations because of COVID-19, are only two entities that will impact data collection critical to future infectious diseases. The era of data-centric computing is here, rate of product change accelerating, storage options expanding, and already digital transformation has taken center stage as a key driver. Outbreaks, epidemics, and pandemics are driving streaming data, larger memory servers, video, artificial intelligence (AI), and machine learning (crucial to guarantee data security), and need for unprecedented “big data and analysis.”^11^ Multi-disciplinary AI questions to the proposed Global Public Health (GPH) Database will be increasingly common, as it should be in pandemics. All these demands require new training and professions. Cost effective and resilient Cloud storage infrastructure can adapt to rapid workload requirements such as transient or spiking demands of world-wide PBM requirements. With the advent of “fast and dense memory,” the intelligent storage can be trained to be largely self-managing, responding to detect anything from race conditions or access to care changes. Harris concludes that the era of data-centric computing is here with the potential to bring top-priority to the demands of the proposed PBM system.^11^

*The Economist* is more detailed in their assessment in managing future pandemics accelerated by population growth and globalization that have markedly increased the spread of infectious diseases:^12^

- Data analysis can pinpoint outbreaks and reveal where they may unfold next.
- Can show where people were moving within 24 hours, right from the initial outbreak, and could distribute that information widely, especially PBMTs.
- Can take advantage of diverse skill sets in tracking new, less-understood pandemics, favoring the proposed multi-disciplinary focus of their teams to include: “physicians, veterinarians, ecologists, data scientists, epidemiologists, environmental health specialists, geographers, designers, and software developers,” to help make sense of raw data and AI requests and critical mobility-related information.^12^
- The WHO concludes that “working together with data firms and health agencies (ie, PBMTs) before a disaster strikes mitigates searching for data at the start of an outbreak, which is already too late, reiterating a multi-stakeholder collaboration archives more than working in isolation.”^12^

## Artificial Intelligence in support of the GPH Database

Expectations were high for AI to help fight COVID-19. However, before AI tools can make an impact, global collaboration and high-quality data and model sharing are needed. *Nature Machine Medicine* emphasized that early warning and alerts, prediction and detection of outbreak of diseases, real-time disease monitoring world-wide, analysis and visualization of spreading trends, prediction of infection rate and infection trend, and rapid decision making to identify the effectiveness of management.^13^ Other authors emphasize challenges that need to be addressed before AI can have a beneficial global impact.^14-16^ They point out that a first challenge is knowing where to start with developing AI tools that can be most effective, which requires close cooperation with practitioners at the health care frontline, those that manage the PDMTs. The best solutions may involve adapting already validated systems rather than building new tools from scratch. Furthermore, Hu, et al described how clinical needs are evolving as the pandemic is moving through different stages, from early detection and anticipation, to containment and mitigation, and finally eradication. During these transitions, the specific types of AI models may need to change too.Ideally, the AI system itself becomes a smart, intelligent, and adaptive system.^13^

## Education and Training for PBM and the GPH Database

The origins of this pandemic can be attributed to climate change and the emergence of complex GPH crises such as climate extremes, biodiversity loss, emergencies of scarcity, rapid unsustainable urbanization, migrant and refugee surges, domestic and international terrorism, the civilianization of war and conflict, and the global rise of resistant antibiotics that have resulted in an unprecedented rise in direct and indirect mortality and morbidity, among others.^17^ In 2019, *The Tohuku Journal of Experimental Medicine* recognized the emergence of complex GPH crises that are beyond the current decision-making and operational capabilities of traditional disaster management and its providers, most of who are community-level practitioners representing every discipline.

The 1930s “disaster cycle” concept described a phase-related approach to meeting the strategic, operational, research, educational, and training components required of disasters; this presents an opportunity for the structured development of a Health Crisis Management Framework to oversee the phase-related strategic and operational requirements for prevention, preparedness, response, recovery, and rehabilitation challenges of major GPH crises.^18^ No longer will physicians, for example, focus on the response phase alone. Future PBMTs will be trained across the entire disaster cycle. Operational knowledge on prevention and preparedness will lessen the number of victims more than any other action. The Japanese Editorial launched academic interest in training multi-disciplinary Health Care Crisis Managers and Scientists to fill the full- and part-time roles required of PBM, PBMTs, and those managing the GPH Database. For example, the university-centered CRIMEDIM (Research Center in Emergency and Disaster Medicine) in Navaro, Italy has worked diligently to offer both Masters and PhD degrees leading to training Health Crisis Managers and Scientists from many contributing disciplines to fill these proposed PBMT positions. A global effort is recommended post-COVID of multi-disciplinary educational programs to help prepare the potential PBMT and GPH Database experts that this study recommends.
